# An Experimental Assessment Using Acoustic Emission Sensors to Effectively Detect Surface Deterioration on Steel Plates

**DOI:** 10.3390/s24196462

**Published:** 2024-10-06

**Authors:** Nikolaos Angelopoulos, Vassilios Kappatos

**Affiliations:** Hellenic Institute of Transport (HIT), Centre for Research and Technology Hellas (CERTH), 57001 Thessaloniki, Greece; vkappatos@certh.gr

**Keywords:** acoustic emission, sensors, corrosion, surface degradation monitoring, structural health monitoring

## Abstract

Acoustic emission (AE) testing is used for the continuous evaluation of structural integrity and the monitoring of damage evolution in structural components and materials. During operation, the environmental and loading conditions of metal structures can result in corrosion and surface wear damage. The early detection of surface degradation flaws is crucial, as they can serve as local stress concentration points, leading to crack initiation and failure. In this work, the effectiveness of AE in monitoring corrosion and surface wear flaw formation was experimentally evaluated. AE sensors were installed on steel test plates during the artificial induction of corrosion and surface wear in order to detect and record the generated AE signals. Corrosion-related AE signals typically exhibit low amplitude, count, and energy values. The direct detection of active corrosion can be challenging in noisy environments, but it can be carried out under certain conditions using dedicated AE sensor groups. Surface-wear-related AE signals exhibit high amplitude, energy, and count values, with long duration values that are associated with wear and grinding conditions. It was found that AE sensors can be utilised to detect corrosion and surface degradation events. The effectiveness of the AE method in detecting surface degradation in noisy environments can be improved by implementing a filtering methodology. This will limit the recording of noise-related signals that can mask out actual surface degradation AE events.

## 1. Introduction

Acoustic emission (AE) testing is a non-invasive method that is based on the detection of elastic stress waves generated during failure and plastic deformation. These waves travel in the material and excite piezoelectric transducers that are placed on the material’s surface, generating an AE signal. Therefore, AE can be used for the early detection of cracking and ongoing failure in a material or structure [1]. Surface degradation on metal surfaces occurs naturally as a result of exposure to the environment and operational conditions during service. A main degradation factor is corrosion, which, if unnoticed or not treated accordingly, can lead to failure and further structural degradation.

The effectiveness of AE in detecting local corrosion and crack growth resulting from stress corrosion cracking has been demonstrated in laboratory conditions and over a wide range of components, metal alloys [2,3] and industrial structures [4]. When corrosion occurs, hydrogen (H_2_) is formed on the cathode areas and produces AE activity. The increased AE activity of such signals is related to accelerated corrosion rates, as was demonstrated in previous studies [5,6,7,8,9]. Moreover, AE can be used to monitor the evolution of corrosion and differentiate between the different pitting stages, which was also discussed by H. Bi et al. [10] and K. Wu et al. [11]. Acoustic Emission can also be used to monitor failure in materials operating in corrosive environments. It was demonstrated that the increased generation of AE signals in corrosive environments is in accordance with the simultaneous reduction in the fatigue limit of materials [9,12]. Additionally, corrosion-induced exfoliation failure can also be monitored using AE [13].

In addition to fatigue damage, sudden failure can occur in structural components, storage tanks, pressure vessels and hulls as a result of corrosion degradation. Operational loads can increase the generation of AE, which facilitates the detection of corrosion areas. Corroded regions are susceptible to crack growth, and their presence can become more pronounced under the influence of applied loads and the weight of the structure. Loads introduced to corroded sections can produce AE activity related to microcracking and the spalling of the corrosion layers [14]. Acoustic emission can be combined with ultrasonic inspection methods for comprehensive in-service structural integrity assessments and corrosion detection for various structures, as presented by A. Anastasopoulos et al. [15].

During service, friction and complex reciprocating loading can result in the formation of surface flaws such as fretting, scoring, and surface metal spalling, which can lead to the formation of microcracks on the base metal. Surface wear and surface degradation can be monitored using AE. Increased AE rates have been reported as the severity of wear on metal surfaces has increased [16,17,18]. In addition, the increase in fretting-wear cycles leads to the generation of AE signals with increased amplitude [19], while the effect of fretting on crack initiation and propagation under fatigue-loading conditions has also been demonstrated [20]. The evolution of friction wear is also reflected in the frequency distribution of resulting AE signals. The frequency spectrum of these signals can include frequency bands that are additional to the main resonant frequencies of the sensors used [21]. Geng et al. [22] reported high intensities of their collected signals at frequency levels of 10–400 kHz, 540–770 kHz, and 850–920 kHz as the wear load increased. The correlation between frequency bands and friction wear was utilised by Baccar and Söffker [23] in order to monitor the evolution of different wear stages. The use of different frequency bands in monitoring and detecting active wear evolution was also demonstrated in previous studies [23,24]. The early detection of surface damage is crucial since microcracking and damage initiation can occur from affected areas, leading to further damage during in-service conditions.

An experimental evaluation of AE for monitoring active corrosion and surface wear is presented in this study. Accelerated corrosion and simulated surface wear were artificially induced on the surface of metal plates under controlled laboratory conditions. The evolution of active corrosion and artificially introduced surface wear was monitored using AE sensors. The main AE signal features and frequency distributions are reported. In order to increase the effectiveness of AE testing for detecting surface wear events, a filtering methodology is discussed, and an appropriate AE sensor selection for the optimal detection of active corrosion and surface flaw formation is proposed.

## 2. Experimental Setup and Procedure

### 2.1. Acoustic Emission Parameter Setup

**Figure 1:**
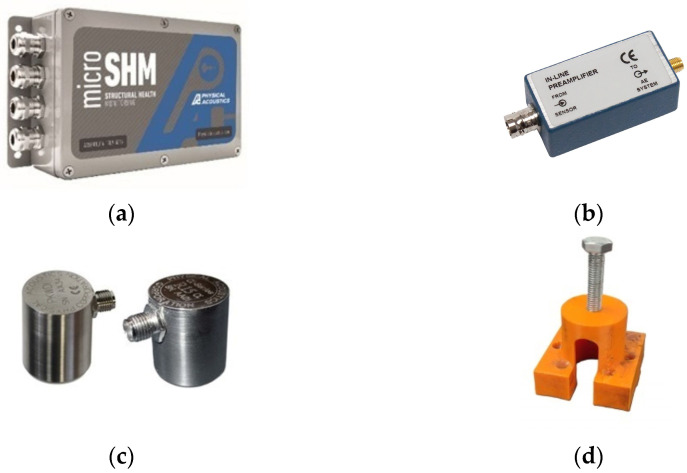
AΕ equipment: (**a**) Mistras Micro SHM AE acquisition system (**b**) In-line preamplifier, (**c**) AE sensors and (**d**) sensor magnetic holder.

In order to evaluate the effectiveness of AE testing in monitoring active corrosion evolution and surface wear damage, modelled surface degradation events were replicated on 30 cm × 30 cm S355 steel plates with a thickness of 10 mm. The artificially induced corrosion and surface wear degradation events were monitored with AE. The AE signals were recorded using a 4-channel Mistras Micro-SHM system. Data acquisition was performed using the Mistras—AE-Win V1.30 software. Due to a lack of former technical knowledge on the AE signal signatures of corrosion and surface degradation, three different types of AE sensors were used in order to assess their effectiveness in detecting corrosion and surface wear. The use of the most effective sensor type was proposed depending on what type of defect needed to be detected. The sensors were a wideband PKWD-I, a resonant R6a and a resonant R15a sensor. The wideband sensor operated within the frequency range of 200–1000 kHz. The R15a sensor operated in the frequency range of 50–400 kHz and had a resonance frequency of 150 kHz, while the R6a sensor had a resonance frequency of 60 kHz and operated in the frequency range of 35–100 kHz. In addition to the AE sensors, preamplifiers were used in order to amplify the electrical output signals. The PKWD-I is an integral sensor and has the preamplifier built-in. For the R15 and R6 sensors, two low power in-line IL-LP preamplifiers were used. The Micro SHM system, sensors, and preamplifiers were acquired from Mistras Group—Hellas. The AE sensors were coupled on the test piece using general purpose grease and were held in place using magnetic holders. The AE equipment is shown in Figure 1, and the AE acquisition parameters for the simulated corrosion and surface wear damage tests are presented in Table 1 and Table 2, respectively.

For all AE tests, the sensors were coupled on the surface of the test piece using general purpose grease as coupling agent and held in place with magnetic holders. The use of coupling agent ensures reliable transmission of AE signals from the surface to the sensor with no signal loss. In addition, pencil lead break tests (PLB) were carried out before each test to verify sensor response and ensure consistent coupling. A PLB test is carried out using a mechanical pencil to break a 0.5 mm 2H graphite lead of approximately 3 mm in length. A plastic guide-ring is used to maintain a constant break angle of 30°. After testing, post-acquisition filtering was applied on the collected AE data to remove any noise-related AE hits and improve the quality of the obtained results. The filtering method was applied on the main signal features of the acquired AE dataset. The filtering parameters are presented on Table 3.

### 2.2. Corrosion

**Figure 2:**
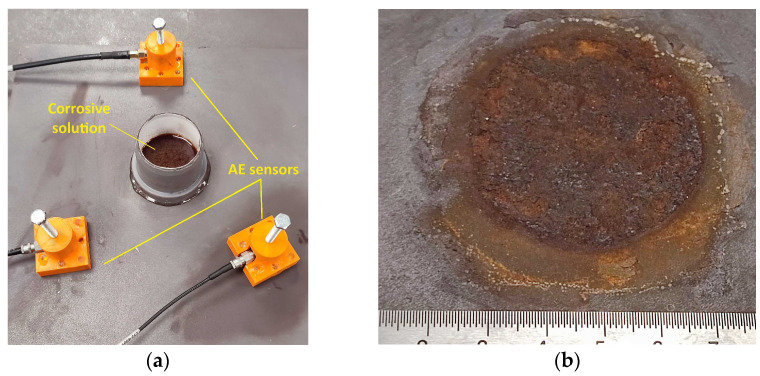
(**a**) Sensor placement around corrosion area; (**b**) corroded area after testing.

For the simulated corrosion tests, a corrosive agent was prepared, consisting of a 50/50 mixture of 3% peroxide solution and 5% acetic acid aqueous solution. A piece of PVC pipe with a diameter of 50mm and a height of 70mm was fixed on the steel plate’s surface in order to contain the corrosive solution, as shown in Figure 2a. The AE sensors were placed at equal distances of 15 cm around the pipe piece. Due to the highly oxidising nature of the solution, the corrosion was rapid enough to allow the sensors to detect AE signals related to active corrosion. After testing, the corrosive solution and the pipe piece were removed from the surface, and rust formation was observed on the plate (Figure 2b). The recorded AE data were analysed, and the main AE signal characteristics of Risetime, Counts to Peak, Duration, Counts, Energy Amplitude, and peak Frequency, along with the frequency distribution of the corrosion-related signals, were determined.

### 2.3. Surface Wear Flaws

**Figure 3:**
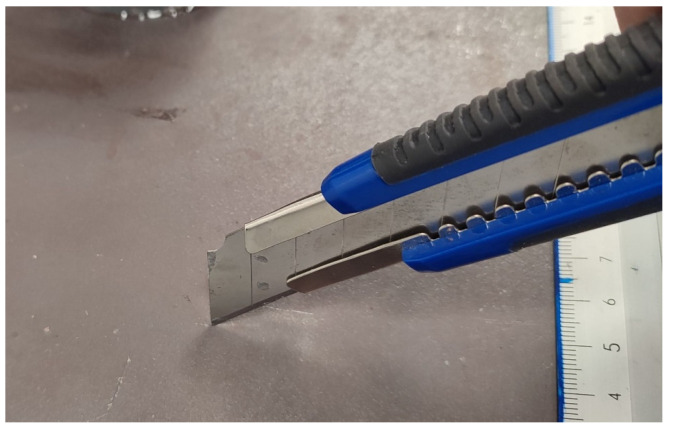
Introduction of scoring marks on the steel plate surface.

**Figure 4:**
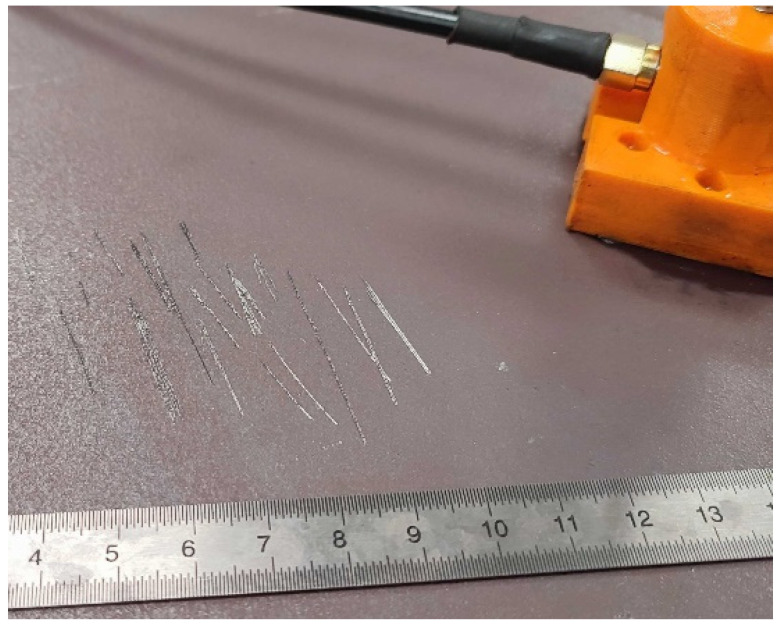
Artificial scoring marks.

To replicate surface wear flaws, scoring marks were introduced on the steel plate’s surface using a utility steel knife blade. As shown in Figure 3, the blade was run and pressed across the surface of the softer S355 steel plate, to create the extensive scoring and scratch marks that are shown in Figure 4. Similarly to the corrosion-simulated tests, the AE sensors were placed around the scoring areas. The artificial surface wear events were generated with caution in order to keep the secondary and additional unwanted noise AE signals at low levels. The recorded AE data were analysed, and the main AE signal characteristics of Risetime, Counts to Peak, Duration, Counts, Energy Amplitude, and peak Frequency, along with the frequency distribution of surface-wear-related signals, were determined and discussed.

## 3. Results

The following section presents the results from the AE testing on steel plates during the accelerated corrosion tests and the introduction of artificial surface wear flaws. The results cover the values of key signal characteristics and the frequency distribution of the recorded AE events. Post-acquisition filtering was applied on the recorded data to remove the AE noise.

### 3.1. Corrosion Tests

**Figure 5:**
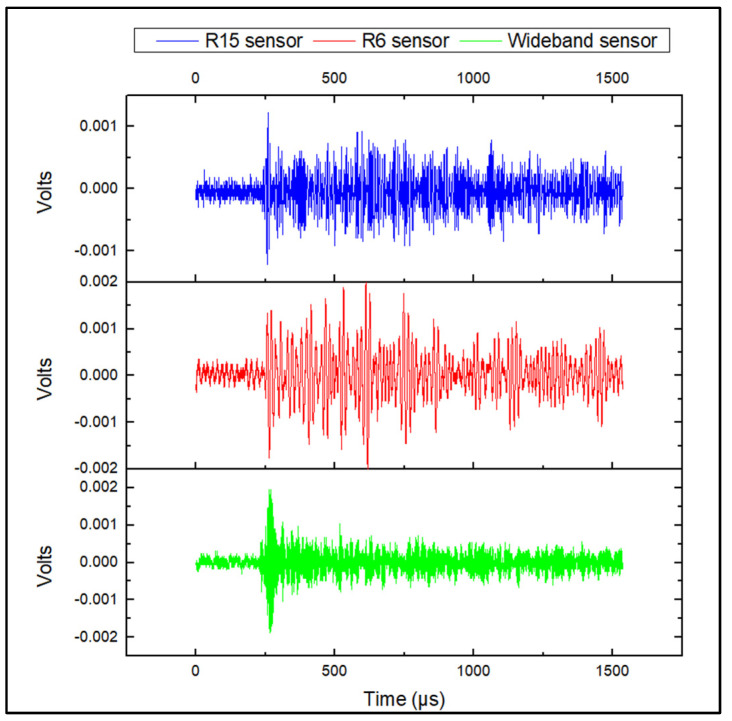
Continuous corrosion-related AE signal.

**Figure 6:**
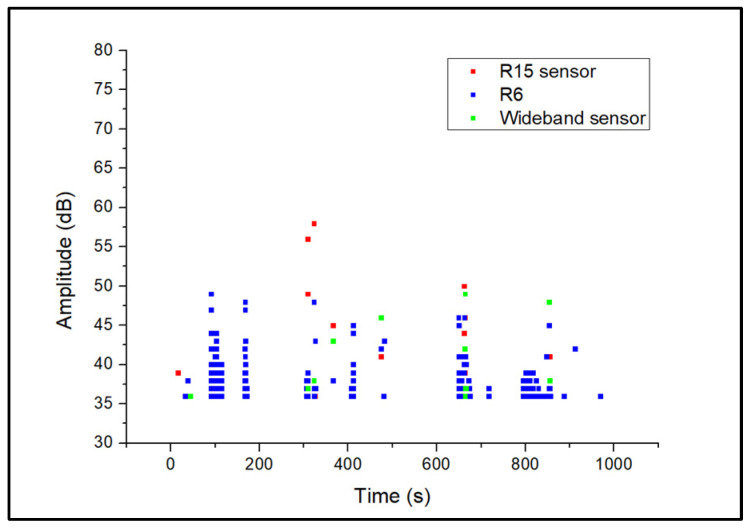
Amplitude scatter distribution of corrosion AE signal.

During acquisition, the generated AE activity was slow and the AE signals, generally appeared as continuous waveforms with low amplitudes. Representative corrosion-related waveforms for each sensor type are shown in Figure 5. The generated AE activity was followed by the formation of hydrogen bubbles in the anode areas of the steel plate, as also demonstrated by previous studies [5,6,7,25,26], where corrosion evolution is followed by the formation and collapse of hydrogen bubbles generating AE activity with low amplitudes within the range of 35–45 dB. A representative amplitude scatter distribution of the corrosion-related AE signals for all sensors is shown in Figure 6. It can be seen that the R6 sensor has recorded clusters of hits that appear at the lowest amplitude range, compared to the R15 and wideband sensors. These signals exhibit the lowest energy, counts, and frequency values and are related to AE noise. The increased detection of such signal types is observed for the R6 sensor due to its low-resonance frequency at 60 kHz, and higher sensitivity at low frequencies below 20–30 kHz, where external noise is typically observed. The R15 and wideband sensors are less sensitive to low-frequency signals and their use can lead to reduced noise signals during acquisition.

A statistic of the main signal characteristics of Risetime, Counts, Counts to Peak, Energy, Duration, Amplitude, and peak frequency, including the resonance frequency for each sensor is presented in Table 4, Table 5 and Table 6, after filtering has been applied on the raw AE data. In addition, in order to limit the influence of extreme values in the dataset, median is used to report the middle value of the reported signal features. Furthermore, the minimum, maximum, and average values are of the aforementioned signal features is reported. 

In general, the energy of the signals is higher in R15 and lower in R6, while the signals from the wideband sensor appear to have shorter risetime and duration values. These differences are attributed to the different responses for each sensor used during testing. In general, for all sensor types, the signals exhibit low amplitude, low count, and counts to the peak, while on average, they have long Risetime and Duration. The R15 sensor is more effective in detecting corrosion-related AE events, as it leads to more prominent signals with higher energy, while the sensor with a resonance frequency at 60 kHz detects more AE signals. However, those are associated with unwanted low energy noise.

**Figure 7:**
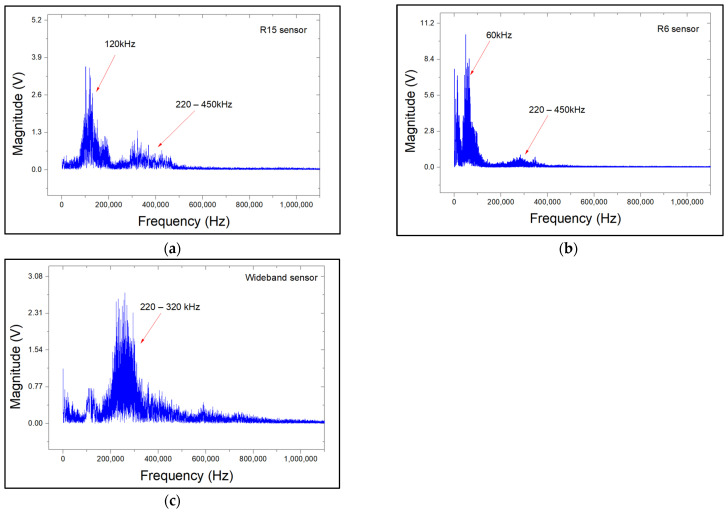
FFT spectrum plots of corrosion-related AE signals for (**a**) R15 sensor, (**b**) R6 sensor, and (**c**) wideband sensors.

After identifying the key signal characteristics, a Fast Fourier Transform (FFT) spectral analysis was performed on corrosion-related signals. The relevant FFT graphs are shown in Figure 7. For all sensor types, apart from the major peaks that correspond to their resonant frequencies, a secondary frequency band in the range of 220–450 kHz is observed. The additional band may indicate the presence of active corrosion. This band is more pronounced for the R15 sensor compared to the R6. This is due to the higher sensitivity of the R6 to low frequencies. However, for the wideband sensor, the aforementioned additional band falls within its peak frequency response range of 200–800 kHz. As a result, the corrosion-related frequency band of 220–450 kHz is masked out by the wideband sensor’s response, and no additional frequency band can be observed in the respective spectrogram

**Figure 8:**
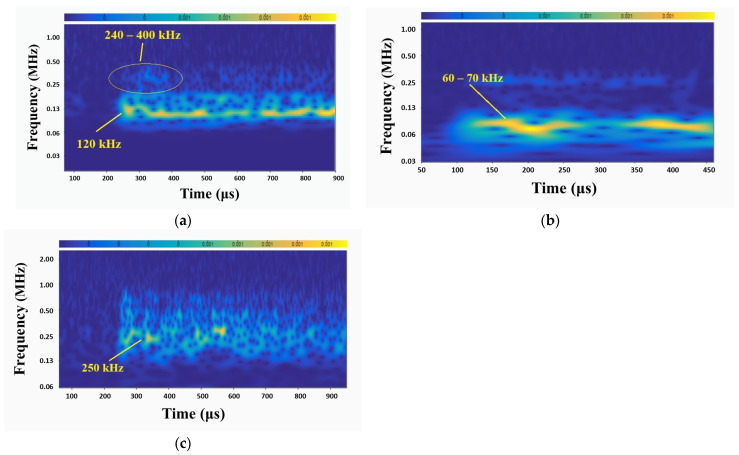
Corrosion-related scalogram of (**a**) R15 sensor, (**b**) R6 sensor, and (**c**) wideband sensor.

In addition to the identification of the main signal features of the corrosion-related signals, scalogram spectral analysis has been carried out. The scalogram analysis method is used to illustrate the evolution of the frequency content of the AE signals over time. Typical scalograms of corrosion-related AE signals for R15, R6, and wideband sensors are shown in Figure 8. In the R15 sensor’s scalogram, a high-intensity area typically appears at 120 kHz, which is close to the sensor’s resonant frequency (150 kHz). Similarly to the FFT graphs, a secondary frequency band that can be associated with corrosion, appears within the range of 240–400 kHz. For the R6 sensor, a high-intensity area appears at the resonant frequency of 60–70 kHz, while an area of lower intensity typically appears around 250 kHz. The scalogram of the wideband sensor exhibits high-intensity frequency regions around 250 kHz. However, a secondary frequency content greater than 500 kHz is also observed, due to the sensor’s wider frequency response. Similarly to the FFT plots, high-intensity areas appear close to the resonant frequency for each sensor, and secondary frequency bands mainly appear in the range of 240–400 kHz.

### 3.2. Surface Wear Tests

**Figure 9:**
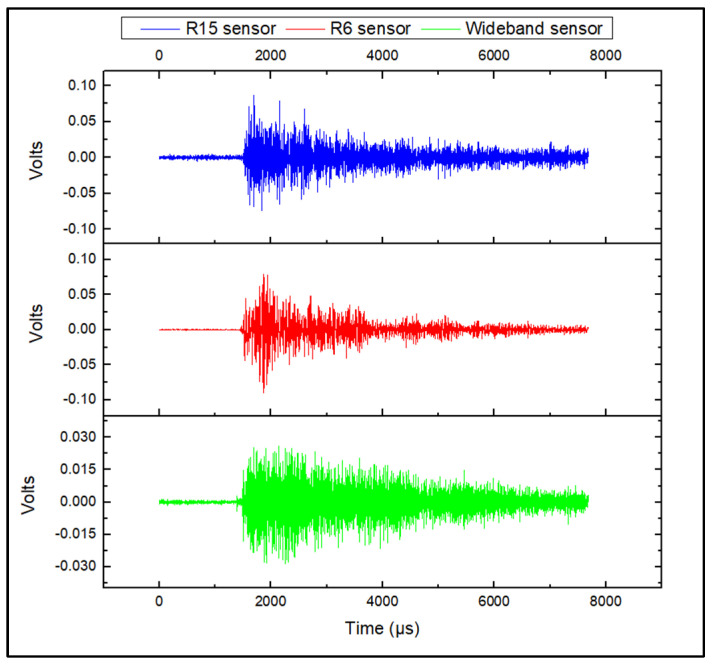
Friction-wear-related AE signal.

**Figure 10:**
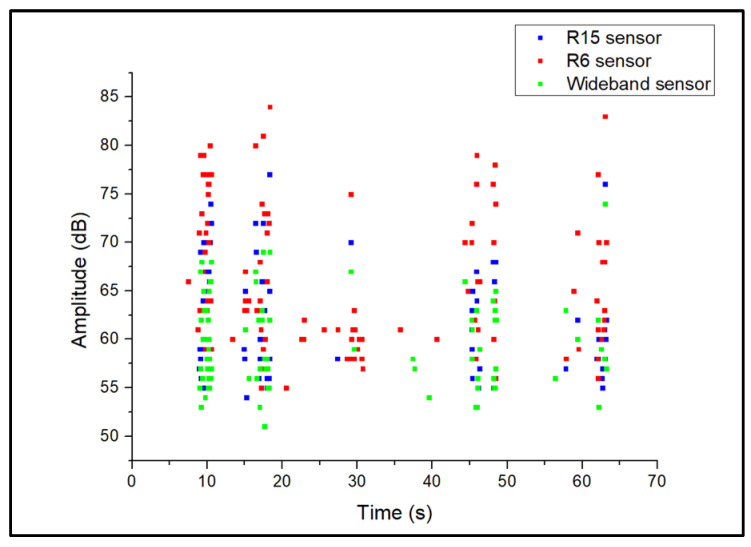
Amplitude scatter distribution of surface wear AE signals for each sensor.

**Figure 11:**
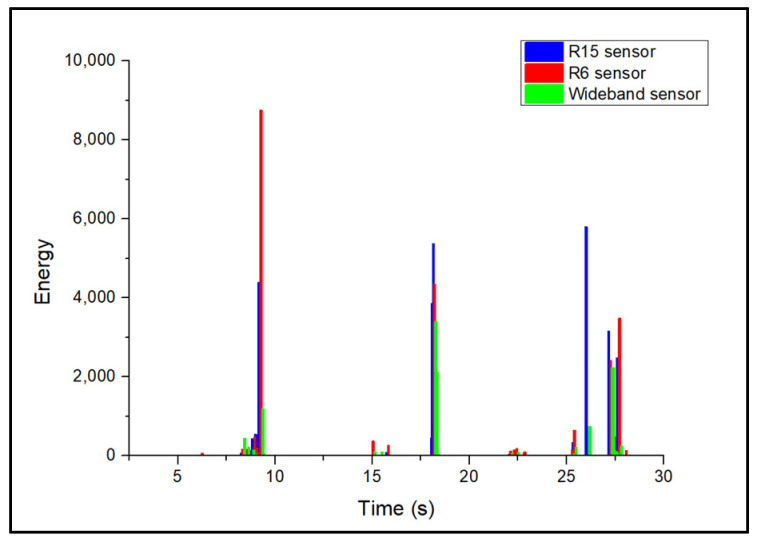
Energy distribution of surface wear AE signals for each sensor.

During the acquisition of AE activity from artificial surface-grinding flaws, the AE signals appeared as long-duration burst-type waveforms. These waveforms had higher amplitudes, duration, and energy values compared to corrosion and noise signal. Typical surface wear AE signals for each sensor are presented in Figure 9. A representative amplitude scatter distribution of surface-wear-related AE signals for all sensors is shown in Figure 10. The introduction of surface wear events is followed by the generation of clusters of AE activity in the range of 50–85 dB. These signals exhibit high energy and long duration values. A typical AE energy distribution of the simulated-surface-wear tests is presented in Figure 11.

Due to its high sensitivity at lower frequencies, the R6 sensor generates signals with higher energy compared to the other two sensor types. Moreover, low values of AE energy can also be observed in Figure 11. These low-amplitude signals are generally associated with friction-induced noise rather than surface wear damage alone. Surface-wear-damage-related AE signals appear at higher amplitudes and typically exhibit higher energy values compared to AE noise.

The main signal characteristics of Risetime, Counts, Counts to Peak Energy, Duration, Amplitude, peak frequency, and resonance frequency for each sensor are presented in Table 7, Table 8 and Table 9. The energy of the signals is generally higher for the R6 and lower for the other two sensors, while the signals from the wideband sensor appear to have shorter risetime and duration values. Moreover, the average peak frequency values are close to the resonance frequency for each sensor. The differences between the values of the main AE signal features for all sensor types are attributed to their different frequency responses.

In addition to the main signals, a few AE signals with considerably greater duration values in the range of 15,000–99,000 μς were generated during the artificial surface wear events. Surface wear damage may be caused by grinding conditions, such as when a ship hull may accidentally collide with rocks causing extensive scoring or scraping to occur on the hull plates. Such abnormal conditions can generate high-intensity AE activity that maintains the signal levels above threshold, keeping the acquisition channel open until the maximum-permitted duration is reached. The features of these signals for each sensor are presented in Table 10, Table 11 and Table 12.

All sensor types generate AE signals with high energy and count values, with sharper rise times in relation to their overall duration and are capable of detecting friction wear when it occurs. Surface wear results from friction events that produce AE signals similar to low-frequency noise. Despite the increased noise levels that can be filtered out, surface-wear-related AE signals can be identified as long-duration bursts with high energy and count values. The use of R6 sensor has led to the recording of a larger number of signals with the exceptionally long duration values compared to the R15 and wideband sensors. As a result, despite the distinctive high-energy signals recorded, the R6 sensor may not be suitable for detecting surface wear as the increased noise levels and long duration signals may render any further signal processing challenging.

Under real-life monitoring situations of structural parts, high levels of environmental noise, and abnormal events, such as tool drops or impacts, can be expected. Such events can generate AE signals with long duration values, which in turn can trigger false alarms. The recording of increased levels of those signals can be avoided by utilising an appropriate front-end filtering strategy during acquisition and with the selection of sensor types that are insensitive to low frequencies often associated with noise.

**Figure 12:**
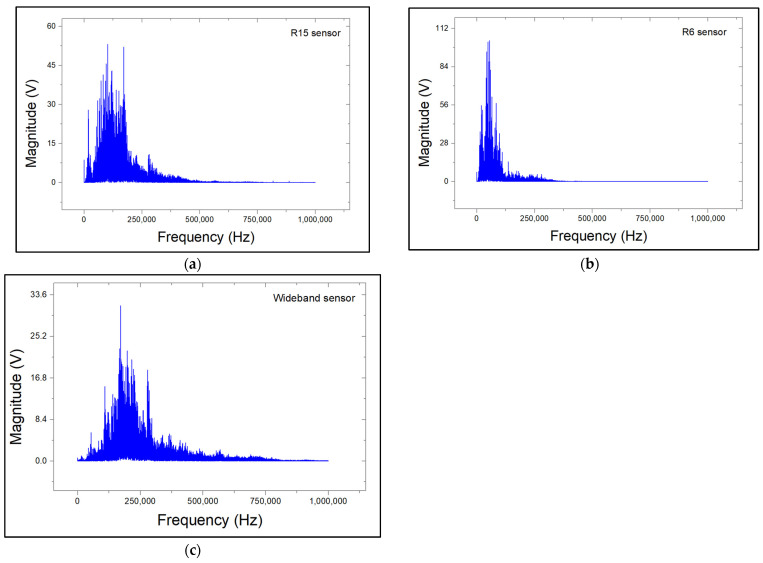
FFT spectrum plots of surface-wear-related AE signals for (**a**) R15 sensor, (**b**) R6 sensor, and (**c**) wideband sensor.

**Figure 13:**
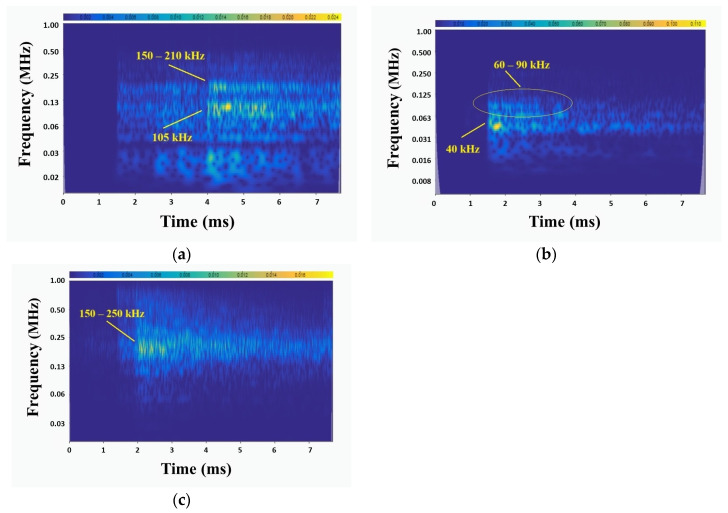
Surface-wear-related scalogram of (**a**) R15 sensor, (**b**) R6 sensor, and (**c**) wideband sensor.

Due to the similarities between surface wear AE signals and noise, no additional frequency bands beyond the main resonance peaks for each sensor can be observed in the FFT plots (Figure 12). Therefore, scalogram spectral analysis was carried out in order to determine the additional frequency content of the wear-related AE signals. Typical scalograms of the recorded signals for R15, R6, and wideband sensors are shown in Figure 13.

For the R15 sensor, high-intensity areas typically appear at 100–120 kHz. In addition to the main frequency, an additional high-intensity area appears in the frequency band of 150–210 kH. The same frequency band is also observed in the scalograms of the wideband sensor. The frequency range of 150–210 kHz that is observed in both of R15 and wideband sensors can be related to surface wear and flaw formation on the metal plate. These signals can be further related to microcracking and microfracture of metal bits that are removed from the steel plate during surface wear events. In the scalograms of the R6 sensor, high-intensity areas mainly appear at 40 kHz, while a lower-intensity area appears in the band of 60–90 kHz. These frequency bands can be related to friction and grinding conditions caused by the hard blade on the softer S355 steel plate, rather than the actual surface flaw formation or surface wear damage.

## 4. Discussion

For the detection of corrosion signals, a sensor resonant at 150 kHz is more effective, as it leads to the recording of more prominent AE signals with higher energies and slightly sharper risetimes. A sensor resonant at 60 kHz can record a greater amount of more signals, but because of its inherent sensitivity to low frequencies, most of them are associated with unwanted noise, with low-energy values. Due to the low sensitivity at low frequencies, the wideband sensor will detect less noise signals, but a significant amount of useful AE signals will not be recorded.

The signal features presented above can also be associated with background noise. Therefore, detecting corrosion in noisy environments can be challenging, as high noise levels during operation can mask-out slow corrosion evolution and give rise to increased environmental AE noise signals. In general, effective monitoring of corrosion areas is indirect. Corroded and pitted areas act as local stress concentration points that can lead to microcrack initiation and crack propagation under operational loads and structural weight. Stress corrosion cracking and crack growth can be effectively monitored using AE, and their detection can lead to the localisation of active corrosion regions.

In order to directly detect corrosion AE signals, specific sensor groups are required, dedicated to corrosion detection. In this case, band-pass filtering can be applied during acquisition. This can limit the recording of noise below the levels of AE corrosion signals and reject noise with higher amplitude counts and energy values. This will allow the detection of signals within the narrow band associated with active corrosion. In addition, further noise reduction can be achieved through AE source localisation between the sensors. In this way, environmental noise or other extraneous events will not be recorded and the effectiveness of the AE system to detect active corrosion can be improved.

The detection of surface degradation formation in noisy operational environments can be challenging as high noise levels can mask the surface damage and give rise to false indications. Therefore, in order to increase the effectiveness of AE in detecting surface wear damage when it occurs, a combination of appropriate sensor selection and filtering strategies is necessary in order to eliminate environmental noise as well as the long duration signals. Further AE noise rejection can be achieved through AE source localization in order to discard any noise and AE activity that falls outside the sensor coverage area.

The selection of a suitable sensor type that is not sensitive to low frequencies (often associated with environmental noise) is essential. Preliminary analysis on noise signals has led to the determination of an initial filtering methodology that is presented in Table 3. In general, AE signals with exceptionally long duration can provide an initial indication of surface wear or major grinding events. A maximum allowable duration filter set at 15,000 μs can significantly reduce the recording of such signals, but some information can be inevitably lost. Nevertheless, this filter maintains maximum allowable duration at relatively high levels, allowing the identification and localisation of surface wear events. Implementing a filtering strategy ensures that a certain level of operational noise and abnormal AE activity is not recorded. This may result in the loss of a limited number of useful signals. However, most surface-wear-related events will be recorded, and the overall performance of AE testing in identifying surface degradation will be improved.

## 5. Conclusions

This work assessed the effectiveness of AE in monitoring corrosion evolution and surface wear deterioration. Corrosion and surface wear degradation were artificially induced on steel plates under controlled laboratory conditions, combined with AE testing. After analysing the recorded AE signals, the following conclusions were drawn:

AE testing can be used to monitor corrosion and surface wear formation. However, the detection of active corrosion in noisy environments can be challenging. Due to the low energy and amplitude values, the overall features of active corrosion AE signals are similar to environmental noise. As a result, high levels of noise can mask-out the detection of active corrosion. Nevertheless, highly corroded areas can still be indirectly detected using AE sensors. Corroded regions are prone to high stress levels under load, leading to subsequent cracking. Therefore, corrosion can be indirectly identified by detecting crack propagation from these areas.

The detection of grinding and surface wear events is more direct due to the generation of AE signals with high energy, amplitude, and duration values. However, high levels of mechanical AE noise and grinding events can still generate unwanted signals with high energy and long duration values. As a result, a filtering methodology that rejects high noise levels needs to be applied during operational conditions.

The effectiveness of AE testing in detecting corrosion and surface wear can be improved through appropriate sensor selection and the development of a filtering methodology that rejects unwanted noise signals. For the detection of active corrosion events, the R15 sensor is more effective at recording corrosion-related events compared to the R6 and wideband. Due to its high sensitivity at lower frequencies, the R6 sensor records high amounts of background noise signals. The wideband sensor records fewer noise signals, but due to its sensitivity at higher frequencies, some corrosion signals may not be detected. For surface wear detection, the R6 sensor is effective at recording surface wear events and signals associated with grinding. However, it picks up increased numbers of friction and grinding-related signals with long duration values, which makes further signal processing challenging. Due to their higher-frequency response, the R15 and wideband sensors provide a better balance between low noise levels and effective recording of surface wear events. In addition, for sensor selection, the use of filtering methodologies can reject environmental noise while allowing effective detection of surface degradation and corrosion events. The use of band pass filtering and AE source location during acquisition can further reduce noise pick-up and improve the performance of AE in detecting corrosion and surface degradation.

The detection of corrosion and surface wear is an important factor, as microcracking and failure are more likely to initiate from the affected areas under load. The detection of such events provides an early warning before failure and cracking become critical, thereby increasing the safety and reliable operation of structural components.

For future work, a correlation will be drawn between the remaining mechanical strength and the evolution of AE activity. This will be based on relevant mathematical formulation and empirical relationships for the prediction of the mechanical behaviour of the tested material.

## Figures and Tables

**Table 1:** Corrosion testing AE acquisition parameters setup.

Acquisition Parameters	Value
Threshold	30 dB
Analogue Filter	20 kHz–1 MHz
Digital Filter	-
Sample Rate	10 MSPS
Pre-Trigger	250 μ
Length	15 k samples
HDT	800 μs
HLT	800 μs
PDT	400 μs
Maximum Duration	99 ms

**Table 2:** Surface wear AE acquisition parameters setup.

Acquisition Parameters	Value
Threshold	45 dB
Analogue Filter	20 kHz–1 MHz
Digital Filter	-
Sample Rate	2 MSPS
Pre-Trigger	1200 μs
Length	10 k samples
HDT	800 μs
HLT	800 μs
PDT	400 μs
Maximum Duration	99 ms

**Table 3:** Post-acquisition filtering parameters.

Signal Feature	Value
Energy	3
Duration	10 μs
Counts	10
Counts to Peak	5

**Table 4:** Main signal characteristics of corrosion-related AE signals of R15 sensor.

R15 Sensor								
	Risetime μs	Counts to Peak	Counts	Energy	Duration μs	Amplitude dB	Peak Frequency kHz	Resonance Frequency kHz
Min	59	6	12	4	1222	37	19	150
Max	3845	61	365	47	9387	53	126	
Average	1059	21	97	15	3875	43	100	
Median	639	14	56	11	3360	43	107	

**Table 5:** Main signal characteristics of corrosion-related AE signals of R6 sensor.

R6 Sensor								
	Risetime μs	Counts to Peak	Counts	Energy	Duration μs	Amplitude dB	Peak Frequency kHz	Resonance Frequency kHz
Min	106	6	11	3	727	35	19	60
Max	6103	116	209	84	9743	60	68	
Average	1393	16	36	12	2940	41	51	
Median	868	12	23	8	2156	40	48	

**Table 6:** Main signal characteristics of corrosion-related AE signals of wideband sensor.

PKWDI Wideband Sensor								
	Risetime μs	Counts to Peak	Counts	Energy	Duration μs	Amplitude dB	Peak Frequency kHz	Resonance Frequency kHz
Min	11	6	22	3	965	38	166	270
Max	4580	60	624	45	9992	55	292	
Average	1092	21	113	10	3395	43	225	
Median	389	15	57	6	2235	41	224	

**Table 7:** Main signal characteristics of surface-wear-related AE signals for R15 sensor.

R15 Sensor								
	Risetime μs	Counts to Peak	Counts	Energy	Duration μs	Amplitude dB	Peak Frequency kHz	Resonance Frequency kHz
Min	53	10	10	7	273	51	21	150
Max	5712	844	844	718	9970	74	175	
Average	658	148	148	105	3328	59	108	
Median	449	99	99	72	2700	59	97	

**Table 8:** Main signal characteristics of surface-wear-related AE signals for R6 sensor.

R6 Sensor								
	Risetime μs	Counts to Peak	Counts	Energy	Duration μs	Amplitude dB	Peak Frequency kHz	Resonance Frequency kHz
Min	81	11	11	11	302	52	17	60
Max	5815	442	442	956	9881	78	97	
Average	765	80	80	131	2972	61	46	
Median	427	57	57	83	2447	60	46	

**Table 9:** Main signal characteristics of surface-wear-related AE signals for wideband sensor.

PKWDI Wideband Sensor								
	Risetime μs	Counts to Peak	Counts	Energy	Duration μs	Amplitude dB	Peak Frequency kHz	Resonance Frequency kHz
Min	19	11	11	4	225	51	125	270
Max	6291	1459	1459	689	9901	75	574	
Average	698	251	251	97	3410	58	212	
Median	470	153	153	66	2877	58	212	

**Table 10:** Signal characteristics of high-intensity surface wear AE signals for R15 sensor.

R15 Sensor								
	Risetime μs	Counts to Peak	Counts	Energy	Duration μs	Amplitude dB	Peak Frequency kHz	Resonance Frequency kHz
Min	100	11	158	217	12,090	57	56	150
Max	38,865	3827	8665	12,369	98,987	90	175	
Average	5330	374	1622	1580	24,254	70	106	
Median	2077	115	996	748	18,041	69	97	

**Table 11:** Signal characteristics of high-intensity surface wear AE signals for R6 sensor.

R6 Sensor								
	Risetime μs	Counts to Peak	Counts	Energy	Duration μs	Amplitude dB	Peak Frequency kHz	Resonance Frequency kHz
Min	146	7	72	216	15,057	55	39	60
Max	61,256	3442	4221	30,608	99,000	99	76	
Average	8268	297	974	2693	30,832	74	47	
Median	4102	115	581	1266	21,664	74	44	

**Table 12:** Signal characteristics of high-intensity surface wear AE signals for wideband sensor.

PKWDI Wideband Sensor								
	Risetime μs	Counts to Peak	Counts	Energy	Duration μs	Amplitude dB	Peak Frequency kHz	Resonance Frequency kHz
Min	145	25	501	254	1024	58	164	270
Max	34,616	2558	6260	3394	45,156	79	226	
Average	4370	452	1904	891	18,786	68	189	
Median	2310	264	1480	588	16,087	67	189	

## Data Availability

Data sharing not available.
